# Improving overall health of children living with HIV through an oral health intervention in Cambodia: study protocol for a randomized controlled trial

**DOI:** 10.1186/s13063-018-3047-z

**Published:** 2018-12-06

**Authors:** Kimiyo Kikuchi, Junko Yasuoka, Sovannary Tuot, Sokunthea Yem, Pheak Chhoun, Sumiyo Okawa, Makoto Murayama, Chantheany Huot, Siyan Yi

**Affiliations:** 1 Institute of Decision Science for a Sustainable Society, Kyushu University, 3-1-1, Maidashi, Higashi-ku, Fukuoka, 812-8582 Japan; 2 Research and Education Center for Prevention of Global Infectious Diseases of Animals, Tokyo University of Agriculture and Technology, Tokyo, Japan; 3 KHANA Center for Population Health Research, Phnom Penh, Cambodia; 4 Department of Community and Global Health, Graduate School of Medicine, the University of Tokyo, Tokyo, Japan; 5 Kawasaki City Dentists Association, Kanagawa, Japan; 6 National Pediatric Hospital, Phnom Penh, Cambodia; 7 Center for Global Health Research, Touro University California, Vallejo, CA USA; 8 Saw Swee Hock School of Public Health, National University of Singapore, Singapore, Singapore

**Keywords:** HIV, Child, Randomized controlled trials, Cambodia, Community-based, Oral care

## Abstract

**Background:**

Currently, the number of children living with HIV is the highest ever. This has led to an increased focus on a healthy life expectancy in this population. Improving oral health status may contribute to improved immunity, which could in turn lead to greater overall health in this population. This study aims to evaluate the effectiveness of an oral health intervention in improving oral health and immune status among children living with HIV in Cambodia.

**Methods:**

A randomized controlled trial will be conducted in Phnom Penh from May 2018 to April 2020. Among 520 dyads of children living with their respective caregivers, half will be randomly allocated to the intervention group and the other half to the control group. Children aged 3–15 years who are currently receiving antiretroviral therapy at the National Pediatric Hospital will be recruited. In addition, 260 HIV-uninfected children (age-matched to the intervention group) will be recruited from the communities. They, together with their caregivers, will comprise the second control group. The main components of the intervention will include oral health education sessions for the children, as well as daily oral self-care under the supervision of their caregivers. The primary study outcome will be the change in oral health status including the number of decayed, missing, or filled permanent teeth, and the secondary outcome will be CD4 count. The effects of the intervention will be assessed by comparing outcome indicators between the children in the intervention and those in the control groups.

**Discussion:**

This trial will investigate the effects of an oral health intervention on the improvement of oral health and immune status among children living with HIV and determine the differences compared with the control groups. This intervention would encourage the promotion of oral health interventions among children living with HIV and thus contribute to delaying the onset of AIDS.

**Trial registration:**

Current Controlled Trials, International Standard Randomized Controlled Trial Number Register, ISRCTN15177479. Registered on 17 January 2018.

**Electronic supplementary material:**

The online version of this article (10.1186/s13063-018-3047-z) contains supplementary material, which is available to authorized users.

## Background

Antiretroviral therapy (ART) has reduced the incidence of new HIV infections worldwide by two thirds in the last 15 years [1]. Accordingly, the lives of many people living with HIV have been saved, and their life expectancies have increased. In high-income countries, the life expectancy of people living with HIV who begin ART at 20 years of age has increased from 36 to 49 years, similar to that of the general population [2]. Among children living with HIV, the mean life expectancy has doubled from 9 to 18 years [3], and the majority of children living with HIV receiving ART in low- and middle-income countries have reached adolescence [4]. These therapeutic advances have yielded the highest-ever number of people living with HIV worldwide, approximately 36.7 million in 2016 [1]. This expansion has led to an increased focus on a healthy life expectancy in this population, although further improvement is required.

Oral health has been identified as a key factor in a healthy life expectancy [5]. In general, a healthy oral status correlates with various parameters of overall health, such as immunity, nutrition status, and cognitive function [6–9]. In addition to dental problems, an unhealthy oral status may affect the healthy physical and mental development of children due to malnutrition, developmental delays, or a lower quality of life [10, 11]. An unhealthy oral status may also predispose one to future lifestyle diseases [12] and may correlate with a high number of disability-adjusted life years [13]. Therefore, the World Health Organization (WHO) recommends initiating oral health care during early childhood [9, 14].

Previous studies have also considered the relationship between oral health and overall health status among adults living with HIV. Periodontal disease was found to correlate with the development of AIDS [15, 16]. A lower immune status has been suggested to correlate with the frequency of oral lesions [17] or caries [18, 19]. HIV-infected people with an advanced stage of immune suppression are often diagnosed with xerostomia due to the reduction of salivary flow. Lower salivary flow hinders the salivary pH level recovery, thus making the oral environment favorable to dental caries [20, 21]. However, studies evaluating the effectiveness of an oral health intervention in improving the overall health status remain scarce among people living with HIV, and a higher level of evidence, such as that generated through a randomized controlled trial, is needed. Particularly, it would be extremely important to determine a causal relationship between oral health and the immunity level, which is generally measured by CD4 count, to contribute to a delayed onset of AIDS. It is particularly important to address these issues in children living with HIV, given the potential effects of poor oral health and malnutrition on maturation to adulthood and the finding that the transition from infection to AIDS and death is compressed in children [22]. Early evaluation and treatment of oral health would therefore be particularly important in terms of the quality of life and longevity in the pediatric population [22].

Furthermore, studies should investigate similarities and differences in the oral health status of children living with HIV relative to that of their HIV-uninfected peers, particularly in response to an oral health intervention. Oral health may have a more prominent effect on overall health status among children living with HIV; this population tends to develop more dental caries [22] and have worse oral lesion statuses [23, 24] relative to their uninfected peers. These discrepancies are partly attributable to the use of ART [22].

Although Cambodia has successfully reduced the incidence of new HIV infections, the prevalence of HIV in the general population remains high (0.6% in 2014) relative to that of other Asian countries [25]. In Cambodia, an estimated 4200 children aged younger than 15 years are currently living with HIV [26]; 87% of these children received ART in 2016 [27]. Concomitantly, pediatric oral health is a critical issue in Cambodia. According to the WHO, the estimated mean number of decayed, missing, or filled teeth (DMFT) among 12-year-olds in Cambodia was 5.50 between 2010 and 2012, compared to the global and Western Pacific Region averages of 1.67 and 1.39, respectively [28]. A study of 6-year-old children conducted in Pailin, Kampong Thom, and Kratie, Cambodia, reported the estimated mean number of DMFT as 7.90 [29]. In another study of school children conducted in the capital city of Phnom Penh, the reported mean DMFT was 2.33 [30]. Further, a study conducted in Siem Reap found that more than half of all children aged 6–16 years had dental caries in their permanent teeth [31], a condition that has been largely attributed to poor tooth brushing habits [31, 32]. Oral health is therefore an emerging public health issue in Cambodia that must be addressed.

The proposed research aims to adopt continual and affordable approaches, such as daily oral care, with an intention to improve the overall health status of children living with HIV in Cambodia. The specific objectives of the study are as follows:

1. To examine the effectiveness of the daily oral care intervention in improving the oral health status of these children, particularly suppressing the increment in the number of DMFT relative to their HIV-infected and HIV-uninfected peers without intervention
2. To examine the effectiveness of the daily oral care intervention on overall health status, particularly including immunity level, nutrition status, and quality of life, relative to their HIV-infected and HIV-uninfected peers without intervention.

## Methods/design

### Study design and sites

This randomized controlled trial will be conducted from May 2018 to April 2020 within the catchment area of the National Pediatric Hospital in the capital city of Phnom Penh. The hospital comprises all diagnostic and treatment departments, including pediatric HIV and dental clinics. This hospital serves as a referral hub for health centers that provide routine pediatric care and treatment, and it is the only health facility to provide pediatric ART in Phnom Penh. See Additional file 1 for the Standard Protocol Items: Recommendations for Interventional Trials (SPIRIT) checklist.

### Study population and selection criteria

**Fig. 1:**
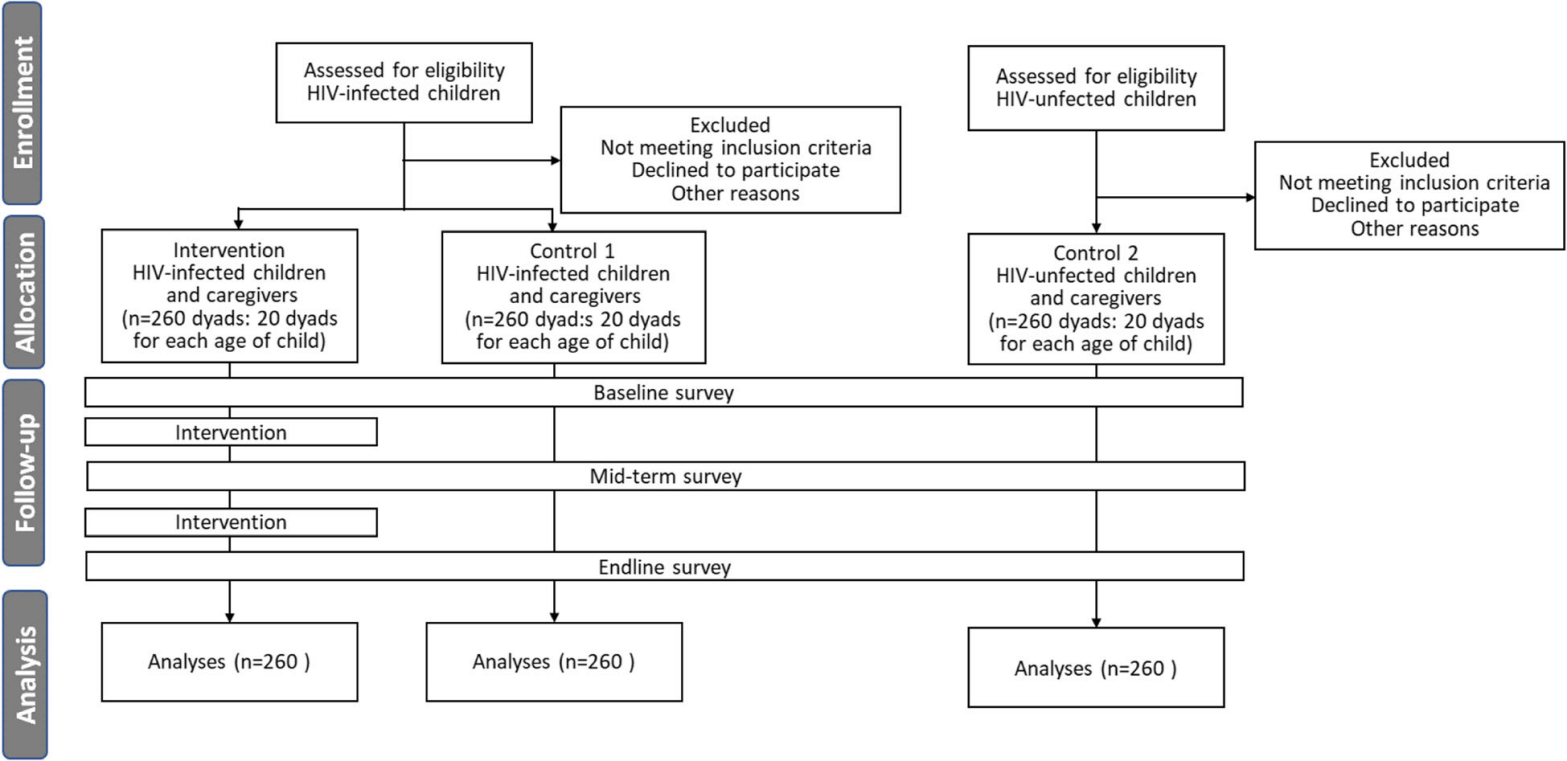
Flow diagram of subject selection

The study will comprise three groups: an intervention arm (group A) and two control arms (groups B and C) (see Fig. 1). The targets of groups A and B will be children living with HIV and their caregivers. Randomization will be performed for groups A and B using a 1:1 allocation protocol. The targets of group C will be HIV-uninfected children and their caregivers, comprising children age-matched with those in group A. Children eligible for groups A and B of the trial must meet all of the following inclusion criteria at randomization: (1) age 3–15 years, (2) have a patient ID at the National Pediatric Hospital, and (3) have received ART for at least 3 months. Children eligible for group C must be aged 3–15 years and must not have been diagnosed with HIV infection. For caregivers in all groups, individuals will be eligible if they are aged ≥ 18 years and self-report as the primary caregiver of the respective child.

### Interventions

Children and caregivers in group A will receive an intervention for 2 years (May 2018–April 2020). The main components of the intervention will comprise oral health education sessions and daily oral self-care. Prior to the intervention, two dental assistants from the National Pediatric Hospital will be appointed to receive a 1-day training course in conducting the oral health education sessions and intervention follow-up. Subsequently, these professionals will disseminate oral health education sessions to the children and caregivers under the supervision of the dentist every 4 months during the 2-year intervention period (in total six sessions for each participant). The initial education session will be provided with the contents presented in Table 1. The dental assistants will also provide the follow-up sessions to the initial session. Each 60-min session, including practice of the oral health procedure, will be attended by groups of 10 child-caregiver pairs. The education session will aim to teach the children and caregivers about correct brushing and the importance of daily oral care. Teaching tools will be developed based on publicly accessible basic oral health education materials which are used in Japan and the USA at school or dental clinics [33, 34].

**Table 1:** Contents of oral health education session

Session 1: Knowledge	
1. What is tooth decay?	
2. Causes and prevention of tooth decay	
Session 2: Practice	
1. Brushing	
2. Flossing	
Session 3: Evaluation of oral health status	
1. Check plaque status using tester	
Session 4: Home-based daily oral care	
1. What one needs to do at home for daily care	
2. Follow-up schedule	

The children and caregivers will practice home-based daily oral care. Each child will receive a toothbrush, two tubes of fluoride-containing toothpaste, and three packs of dental floss at every education session. During the session, the children will be advised to brush daily after breakfast, lunch, and dinner by themselves or with the help of a caregiver at home. To maximize the intervention adherence, the children will receive a daily record notebook to keep their daily oral care practice. In the notebook, a short oral care guide is presented to which the children and caregivers can refer any time at home. Also, the children affix a seal to the daily record notebook after each brushing and evaluate their weekly oral health practice on the checklist. The intervention will be discontinued at any time should the participant request it. Participants can receive any concomitant care and intervention during the trial.

The participants of groups B and C (randomized control arms) will not receive specific interventions for oral health management. Participants in all groups can receive any type of dental care as needed at a clinic, but they will be responsible for any expenditure.

### Follow-up

After each 4 months follow-up education session by the dental assistants, children and caregivers in the intervention group will meet the research assistants for monitoring of the children’s daily oral self-care performance. The research assistants will note the number of seals placed in each child’s daily record notebook and the number of checks in the weekly checklist. Then, the children will be asked questions regarding their health status in accordance with the overall/oral health-related quality of life questionnaires, and their height and weight will be measured. Only the CD4 counts will be measured once a year.

### Outcome measures

**Fig. 2:**
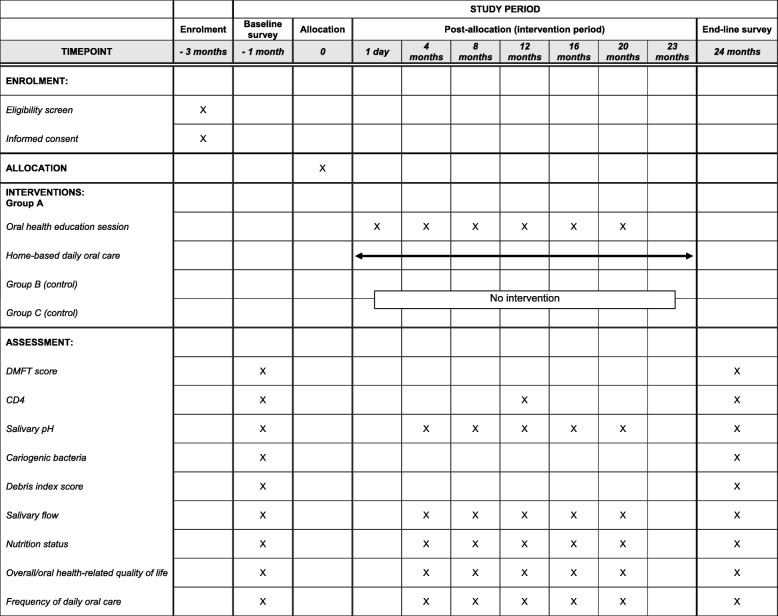
Participant timeline

The primary study outcome will be the change in oral health status, which will be measured according to the children’s individual DMFT. The secondary outcome will be the CD4 count. The other important outcomes include the salivary pH level, number of cariogenic bacteria, debris index score, salivary flow, nutrition status (height and weight), overall or oral health-related quality of life, and frequency of daily oral care. Details are presented in the data collection section and Fig. 2.

### Participant timeline

The efficacy of the intervention will be evaluated by comparing the intervention and control groups using baseline and endline (2 years after implementing the intervention) survey data. The details are described in Fig. 2.

### Sample size

We calculated the sample size for the intervention and control groups as follows. We estimated that the intervention may suppress the increment of DMFT for around 17% based on the results of a previous study conducted in Cambodia among 8000 uninfected school children in five provinces. In that study, increments of DMFT of intervention and control groups were 0.82 and 0.99, respectively, after a 2-year intervention [35]. According to our situation analysis survey, which was conducted at the same study site in February 2017, for children living with HIV aged 8–15, the mean DMFT was 4.34 (standard deviation 3.3). We estimate that the intervention may suppress the increment of DMFT by 0.7 point, which equates to the 17% decrease in the mean DMFT. To detect this difference in our intervention group, at a power of 80% and a 5% significance level, 260 samples will be required for each group with a potential attrition of approximately 10%. In total, 780 pairs will be required for the three groups.

### Recruitment

Recruitment will be conducted via a multiple-step process. First, children living with HIV aged 3–15 years will be identified from the patient lists of the National Pediatric Hospital. According to the list, 1113 children are eligible based on the study criteria. Twenty children in each age category from 3 to 15 years old will be randomly selected from each list to yield 260 children each for groups A and B. Second, the same patient lists will be used to identify one district wherein the largest number of children living with HIV reside. The children in group C will be selected from that district, using lists of residents aged 3–15 years that have been compiled by a local non-governmental organization, in collaboration with the local authorities. Twenty children per age category from 3 to 15 years will be randomly selected from those lists to yield 260 children for group C. The children deemed eligible for the intervention will be provided with information about the study, and the research assistants will seek an expression of interest from the caregivers and the children. They will be contacted by the research assistants to arrange a suitable time for the baseline survey. Prior to the survey, the research assistants will obtain their written informed consent.

### Randomization

After the baseline survey, the children living with HIV will be randomly allocated to an intervention group in a 1:1 ratio. A computerized algorithm will be used to generate a randomization scheme to assign participants to the study arms. The allocation will be implemented by a data analyst who is not a primary member of the study team. A member of the research team will inform the participants of their allocation. The research assistants, principal investigator, and primary study team members will be blinded to the group allocations until after the participants have completed the baseline assessment.

### Data collection

The effects of the intervention will be assessed by comparing key outcome indicators among children in the intervention and the control groups. Comparisons between groups will be performed by measuring the outcome indicators at baseline and endline (see definitions in the participant timeline section) in the intervention and the two control groups. The surveys will be administered to children and caregivers using a structured questionnaire. We will also conduct oral health and nutritional status assessments of the children in all groups and investigate the CD4 counts and registered clinical data of children living with HIV in groups A and B.

Six research assistants will receive a 1-day training session to ensure their understanding of the questionnaires. After the training, they will administer the questionnaires to 10 children as a pre-test. Two dentists and two dental assistants will receive a half-day training session for oral health data collection. After the training, they will collect oral health data from five children for a pre-test. The data collected for pre-test will not be included in the main analysis.

#### Questionnaires

Separate questionnaires will be developed for the children and caregivers. The questionnaire for children will be administered only to children aged 6–15 years and will solicit information regarding socio-demographic characteristics, HIV symptoms, oral health knowledge, attitudes and perceptions, and oral health-related/overall health-related quality of life. The questionnaire for caregivers will solicit information regarding soci-demographic characteristics, oral health knowledge, attitudes and perception, the child’s adherence to ART, and dietary diversity. The questionnaires will be developed based on existing tools adapted from previous studies [36–38]. To increase the validity of these tools, the questionnaires will first be developed in English and translated into Khmer. The accuracy of the translations and reliability of the contents will be reviewed by three native Khmer-speaking health specialists in the research team. Additionally, the questionnaires will be pre-tested, and inappropriate questions will be revised accordingly.

The quality of life will be measured both for overall health and oral health. Overall health-related quality of life will be measured using the Pediatric Quality of Life Inventory 4.0 (PedsQL ™ 4.0) [36]. This inventory is validated for HIV-infected children. The PedsQL ™ 4.0 is composed of 23 question items across four subscales: physical, emotional, social, and school functioning. The responses for each item are presented in the form of a 5-point response scale: ”never” = 0, ”almost never” = 1, ”sometimes” = 2, ”often” = 3, and ”almost always” = 4. Items will be reversed scored and linearly transformed to a 0–100 scale: 0 = 100, 1 = 75, 2 = 50, 4 = 0. The score is calculated based on the sum of all the items over the number of items answered on all the scales. The total score ranges from 0 to 100; a higher score indicates a better overall health-related quality of life. Cronbach’s alpha was 0.77.

Oral health-related quality of life will be measured using the Child Perceptions Questionnaire, which was validated in Cambodia [39, 40]. It consists of 16 question items across four subscales: oral symptoms, functional limitations, emotional well-being, and social well-being. The responses of each item are also presented in the form of a 5-point response scale: ”never” = 0, ”once or twice” = 1, ”sometimes” = 2, ”often” = 3, and ”every day or almost every day” = 4. The total score ranges from 0 to 64. A higher score indicates worse oral health-related quality of life. Cronbach’s alpha was 0.81.

#### Oral health status

The children’s oral health status will be assessed by examining the number of DMFT, the debris index, salivary pH, salivary flow, and number of cariogenic bacteria. The debris index will be calculated based on the degree of dental plaque. Dental plaque will be evaluated by colorizing the children’s teeth with a plaque test kit. The salivary pH will be assessed using CAT21Buf© (Morita, Osaka, Japan). The salivary flow will be measured as the total quantity of saliva produced in 3 min with stimulation from chewing gum. CariScreen© (CariFree, Albany, OR, USA) will be used to count cariogenic bacteria in saliva collected from the front teeth. All the oral health data will be collected by two dentists with the help of two dental assistants from the National Pediatric Hospital.

#### Nutrition status

To assess the children’s nutrition status, their body weight (in kilograms) and height (in centimeters) will be measured. Electronic scales and a stadiometer, which calibrate to 0.1 kg and 0.1 cm, respectively, will be used. Data will be collected by the nursing staff of the National Pediatric Hospital. The Z-scores of weight for age, height for age, and body mass index (BMI) for age will be calculated using the WHO tool AnthroPlus (available at http://www.who.int/growthref/tools/en/).

#### Clinical records

The clinical records of children living with HIV will be collected by research assistants from the registered documents of the National Pediatric Hospital. The following data will be recorded for the study purposes: CD4 count, history of opportunistic infections, ART regimen, and duration of ART use.

### Data management

The questionnaire responses and follow-up data will be entered directly into Open Data Kit 2.0 (ODK; available at https://opendatakit.org/use/2_0_tools/) by the research assistants. Oral health, weight, height, CD4 counts, and other registered clinical data will be collected by research assistants using a paper-based form. Supervisors will check the data collected during visits for accuracy and completion. Collected paper-based data will be entered electronically by the data management assistant. This will be done at the National Pediatric Hospital. Verification checks will be performed to correct discrepancies in the records. The original forms will be kept on file at the National Pediatric Hospital.

### Statistical analyses

Baseline and endline surveys will be conducted to assess changes in each outcome in both the intervention and control groups. To minimize overestimation of intervention impact, all intervention outcomes will be estimated with an intention-to-treat analysis. Initially, survey data will be stratified by different age groups: 3–5 years old, 6–11 years old, and 12–15 years old. Then, the data will be descriptively analyzed to assess the distributions of various factors related to the children and caregivers under the study. Next, bivariate analyses will be conducted to assess the correlations of each oral health indicator (DMFT, debris index, bacterial number, salivary pH, salivary flow, oral-health related quality of life) with different variables, using the chi-square test (or Fisher’s exact test when a count is smaller than five) for categorical variables, and Student’s *t* test for continuous variables. Then, we will apply a generalized estimating equation model [41] adjusted for basic demographic characteristics (e.g., sex) and factors identified as significantly associated with the outcome in the bivariate analyses. The statistical significance will be set at a *p* value < 0.05. All data analyses will be performed using IBM SPSS, version 24.0 (SPSS Inc., Chicago, IL, USA).

### Monitoring

An intervention monitoring team will be formed as part of the research team. This team is responsible for monitoring of all implementation activities. All adverse events and unintended effects of intervention will be reported to the monitoring team. Access to all monitoring- related information will be limited to the monitoring team members.

## Ethics and dissemination

### Ethical considerations

This study has been approved by the National Ethics Committee for Health Research, Ministry of Health, Cambodia (289NECHR).

### Individual consent

The research assistants will obtain informed consent from all caregivers prior to the intervention, and assent to participate in the study will be obtained from all children. The caregiver’s consent will be recorded through a signature or thumbprint. Participants can withdraw from the study for any reason, and this will not affect the normal services received at any of the health facilities.

### Post-trial care

The intervention will be introduced to the control groups’ participants immediately when a positive impact is identified.

### Confidentiality of information

All information obtained through this study will be confidential. Access to information will be limited to research assistants for conducting interview and data entry management staff during the research period. Study records will be identified only by means of study identification numbers.

### Dissemination

The findings of this study will be disseminated through peer-reviewed journals and international conferences. Important protocol modifications will be communicated to the research ethics committee and trial registry.

## Discussion

Here, we have described a study protocol for a randomized controlled trial to evaluate the effectiveness of an oral health intervention program in improving oral health status, which in turn will improve the immunity and overall health status of children living with HIV in Phnom Penh, Cambodia. This trial aims to investigate how the improvement of oral health status can contribute to the improvement of overall health status in a cohort of children living with HIV, compared with their HIV-infected and HIV-uninfected counterparts who do not undergo the oral health intervention. If proven effective, this oral health intervention could be suggested as another means to delay the onset of AIDS in this population and would lead to the further encouragement of oral health interventions for children living with HIV.

However, the proposed study has some limitations. First, the intervention will target children living with HIV receiving ART. Therefore, we will not be able to distinguish between the effects of ART and that of the oral health intervention on CD4 count or oral health status. Second, samples will be recruited only at one national hospital, and therefore this is a potential source of sampling bias. However, this study has the potential to provide strong evidence regarding the effectiveness of an oral health intervention in an area concentrated with children with HIV receiving ART.

### Trial status

The trial was registered in the International Standard Randomized Controlled Trial Number Register on 17 January 2018 (ISRCTN15177479) (http://www.isrctn.com/ISRCTN15177479). Recruitment for intervention commences in February 2018 and will continue until April 2020.

## Additional file

Additional file 1: SPIRIT checklist. (DOC 123 kb)

## Abbreviations

AIDS: Acquired immune deficiency syndrome
ART: Antiretroviral therapy
DMFT: Decayed, missing, or filled teeth
HIV: Human immunodeficiency virus
PedsQL: Pediatric Quality of Life Inventory
